# Supplementary material for: High-throughput cell and spheroid mechanics in virtual fluidic channels
Source: Nat Commun. 2020 May 4;11:2190. doi: 10.1038/s41467-020-15813-9 (PMC7198589; doi:10.1038/s41467-020-15813-9)
Supplement: Supplementary file 1 — Supplementary Information [file 41467_2020_15813_MOESM1_ESM.pdf]

Supplementary Information

High-throughput cell and spheroid mechanics in virtual fluidic channels

Panhwar *et al.*

A

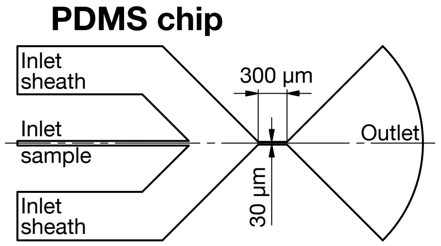

B

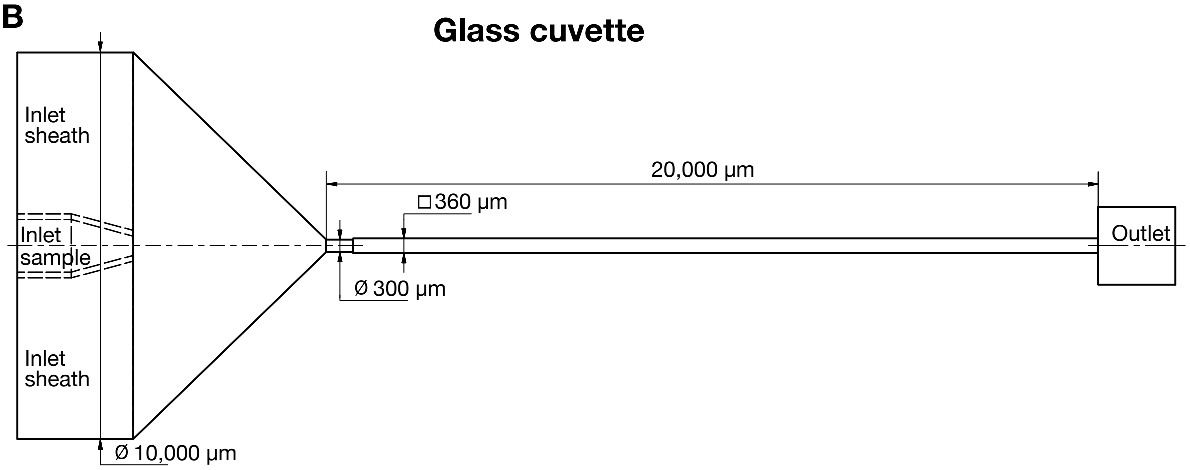

Supplementary Figure 1: Technical drawings of fluidic devices. A 2D projection of a microfluidic chip made of polydimethylsiloxane (PDMS) using soft lithography. **B** Glass cuvette used in a flow cytometer.

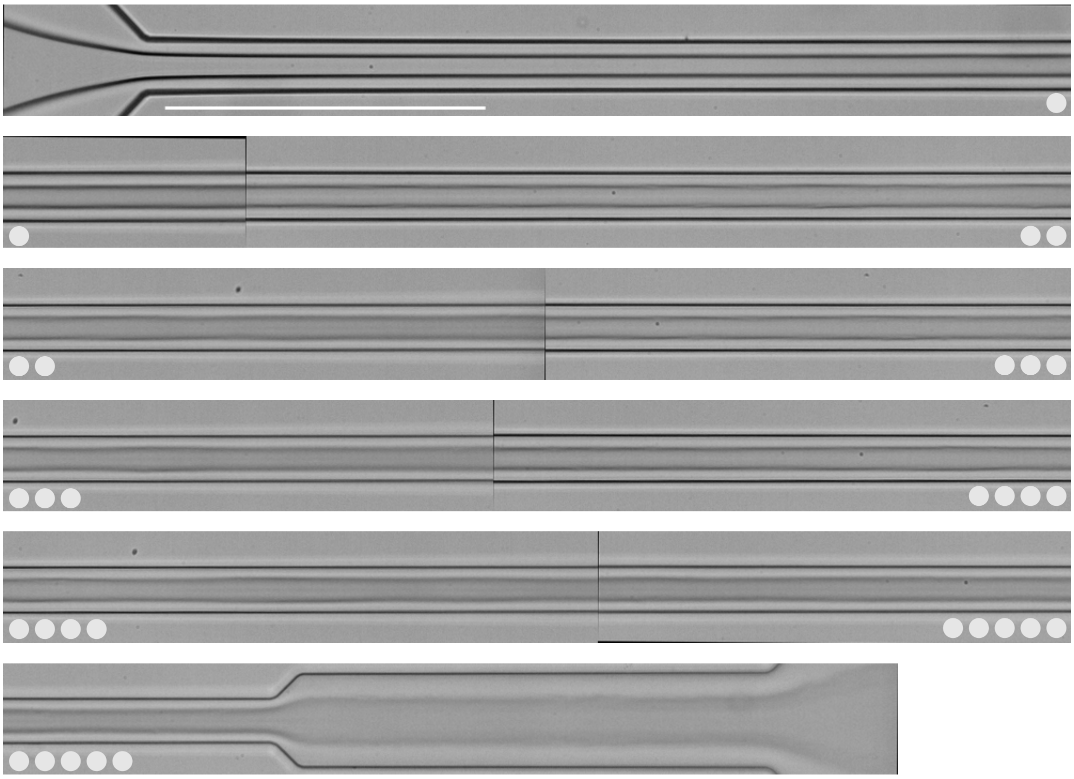

Supplementary Figure 2: Virtual channel in a long PDMS constriction. Stitched image of virtual channel formed by co-flowing sample (57 µM MC, Q_{sa} = 120 nl s^{-1}) and sheath (5 mM PEG40000, Q_{sh} = 70 nl s^{-1}) inside a PDMS constriction of 2 mm length. Video was acquired at 256 frames per second and consists of 1,280 frames in total. The very wide field of view required refocusing in height. Recording of time series has been repeated three times. Scale bar 100 µm.

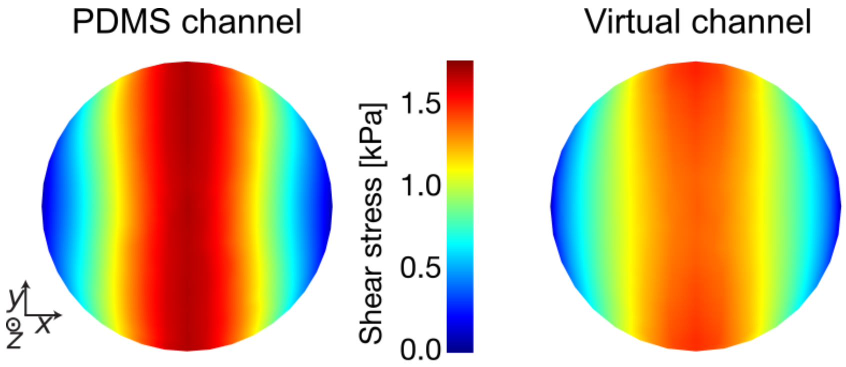

Supplementary Figure 3: Shear stress on cell surface in PDMS and virtual fluidic channel. Finite element method simulations were performed on full geometries of a 20 µm x 20 µm PDMS chip (left) and a virtual channel of 21 µm width and 30 µm height inside a 30 µm x 30 µm PDMS chip (right). Sample solution was MC (114 µM) and cells were modelled as spheres with a radius of 6.6 µm. Flow rates have been adjusted to Q_{sa} = 40 nl s^{-1} (PDMS channel) yielding a mean shear stress on cell surface of 970 Pa and Q_{sa} = 90 nl s^{-1} (virtual channel) yielding a mean shear stress of 907 Pa.

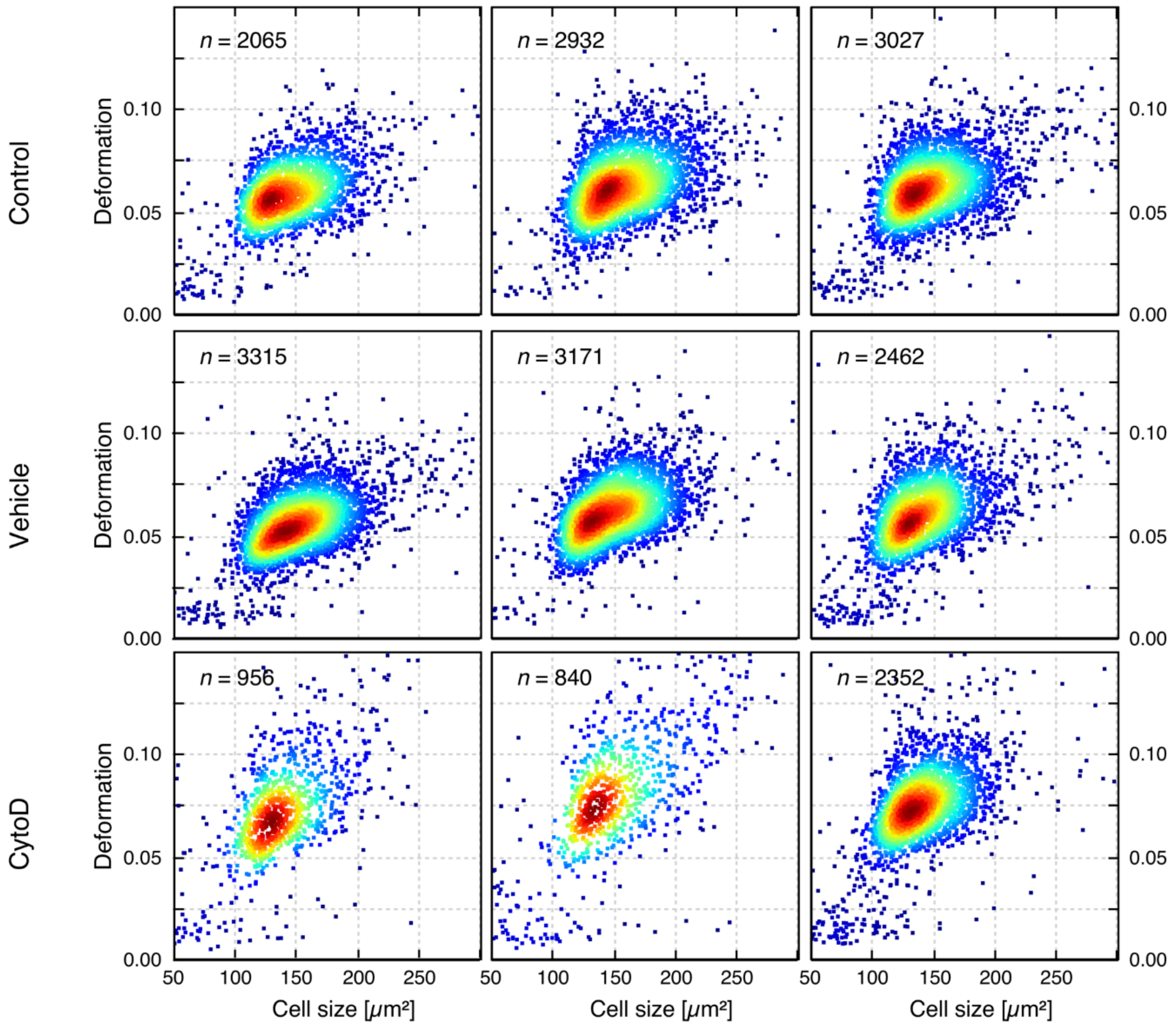

Supplementary Figure 4: Real-time deformability cytometry on HL60 cells inside a PDMS chip. Scatter plots of cell size and deformation measured inside a PDMS chip of 20 μm x 20 μm cross-section at a total flow rate of Q_{tot} = 40 nl s^{-1} (sample MC (57 μM), Q_{sa} = 10 nl s^{-1}; sheath MC (57 μM), Q_{sh} = 30 nl s^{-1}). The columns show three biological measurements in MC (control), under addition of DMSO (0.25% (v/v), vehicle), and exposure to CytoD (1 μM).

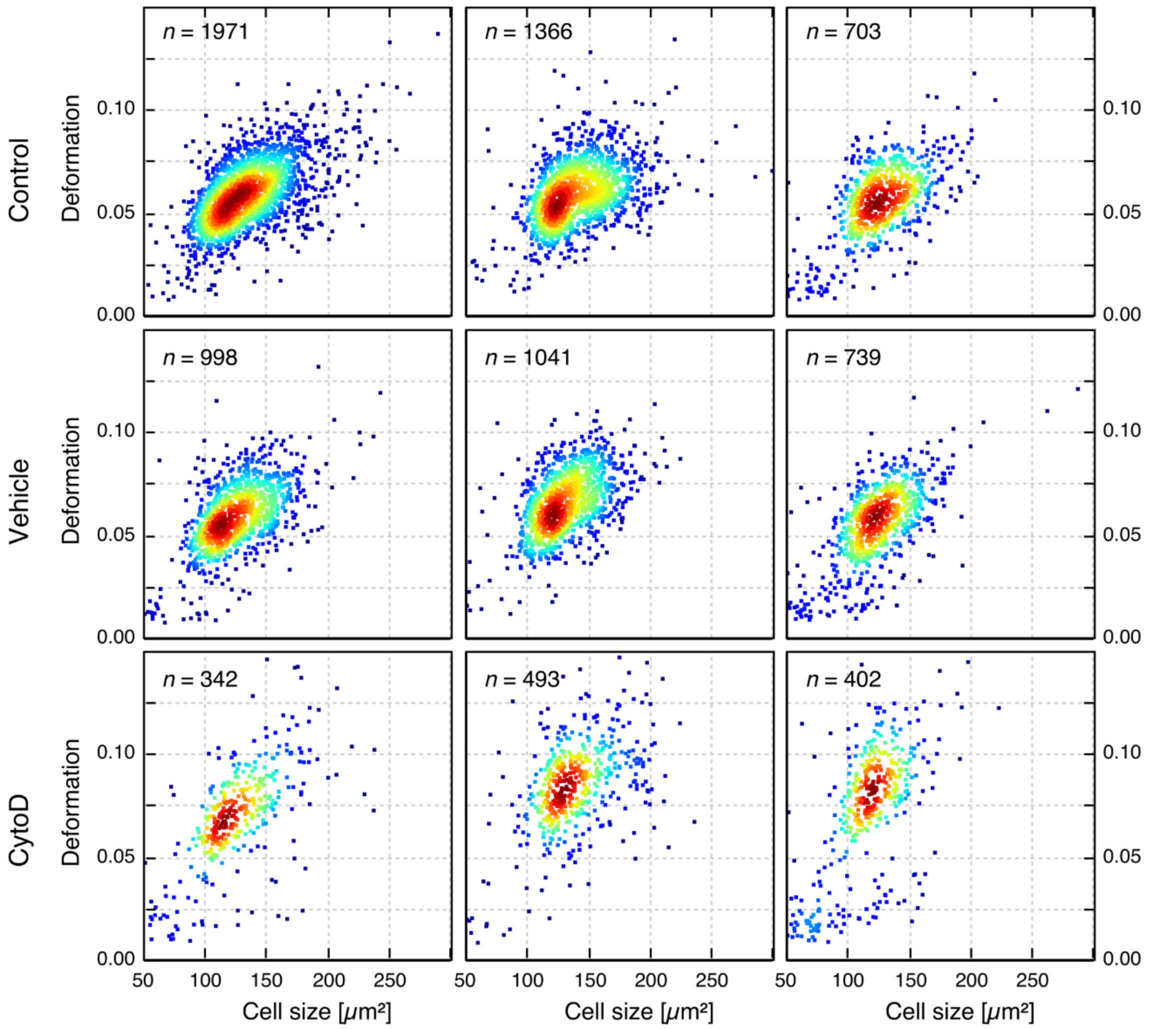

Supplementary Figure 5: Real-time deformability cytometry on HL60 cells using virtual channels in PDMS chip. Scatter plots of cell size and deformation measured inside a 21 µm x 30 µm virtual channel in a 30 µm x 30µm PDMS chip at total flow rate Q_{tot} = 94 nl s^{-1} (sample MC (57 µM), Q_{sa} = 90 nl s^{-1}; sheath PEG8000 (50 mM), Q_{sh} = 4 nl s^{-1}). The columns show three biological measurements each in MC (control), under addition of DMSO (0.25% (v/v), vehicle), and exposure to CytoD (1 µM).

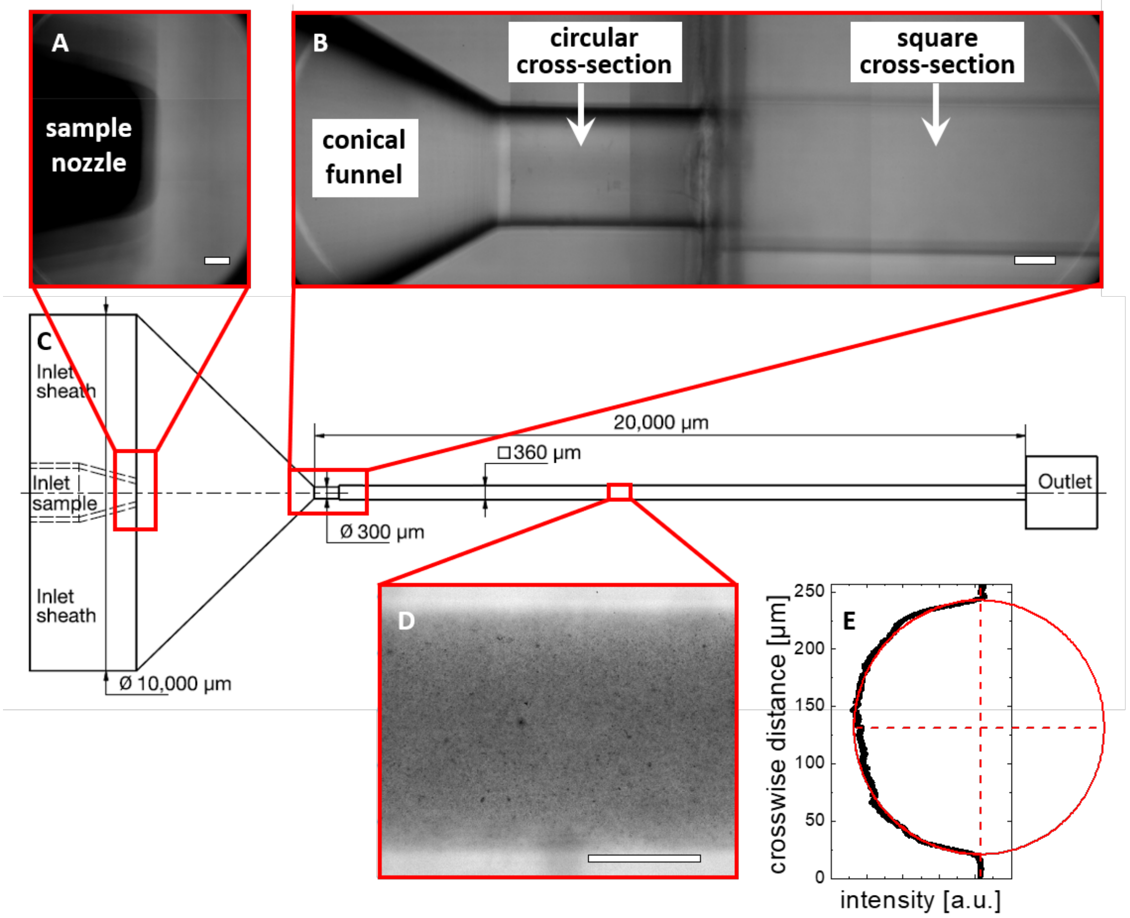

Supplementary Figure 6: Close-up images of flow cytometer glass-cuvette and geometry of the virtual fluidic channel. A Microscopy image of the sample inlet nozzle. **B** Stitched microscopy images of the transition from merging funnel to cuvette channel. **C** Technical drawing of flow cytometer glass cuvette with markings of the pictured regions (red rectangles). **D** Microscopy image of virtual channel formed by co-flowing sample (H_2O, $Q_{sa} = 500$ nl s^{-1}) and sheath (50 mM PEG8000, $Q_{sh} = 1000$ nl s^{-1}) inside the cuvette channel. Water-soluble dye added to the sample flow provides additional contrast due to light absorption. Width of virtual channel $w = 228$ µm. **E** Intensity profile across the fluidic virtual channel. Red circle and crosshairs serve as guide to the eye. Scale bar 100 µm.

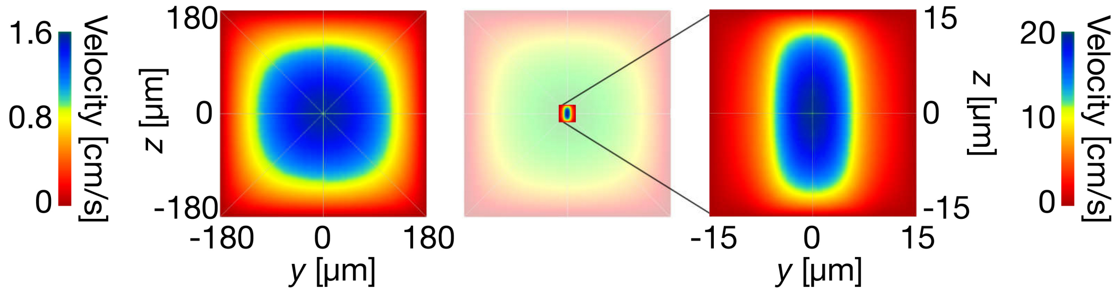

Supplementary Figure 7: Velocity profiles inside glass cuvette and PDMS chip. Finite element method simulations were performed on full geometries of glass cuvette (left) and PDMS chip (right) and yield velocity profiles for the respective cross-section. For the glass cuvette, the cross-section is indicated in Fig. 3B, and the data are shown for sample MC (114 µM) at Q_{sa} = 20 nl s^{-1}, sheath PEG40000 (5 mM) at Q_{sh} = 1,000 nl s^{-1}. For the microfluidic chip the presented cross-section is indicated in Fig. 1A bottom inset and located before the constriction outlet (grey rectangle), and data are shown for sample MC (57 µM) at Q_{sa} = 48 nl s^{-1}, sheath PEG8000 (50 mM) at Q_{sh} = 8 nl s^{-1}. The centre panel compares both cross-sections to scale. Color code indicates velocity magnitude.

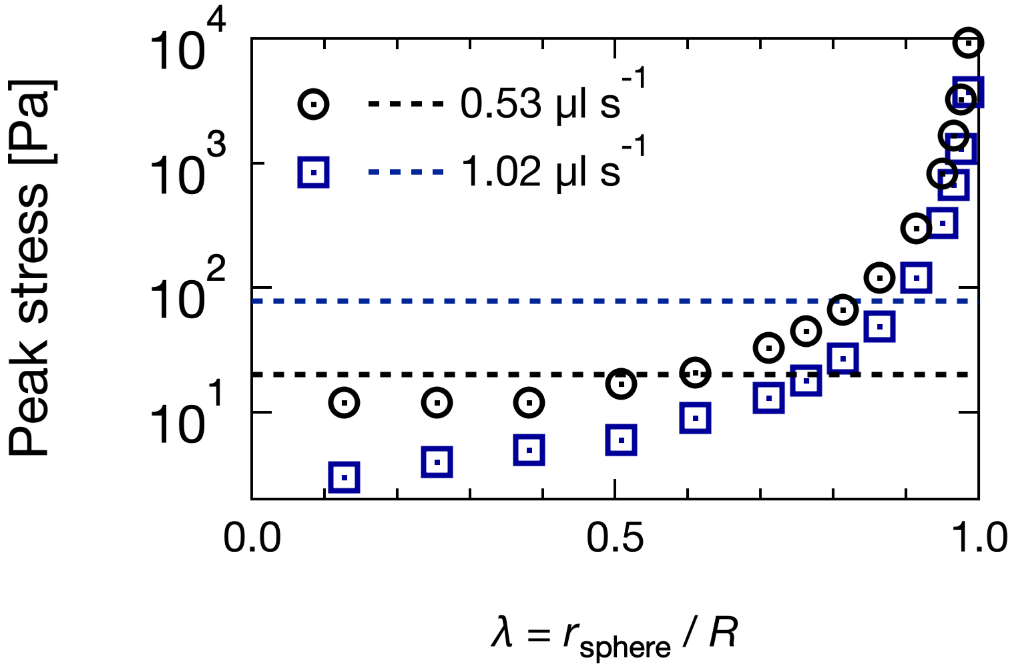

Supplementary Figure 8: Peak shear stress on cell surface as function of cross-sectional coverage in a cylindrical fluidic channel. Finite element method simulations were performed on full geometries of a cylinder (radius R) filled with a Newtonian media of viscosities 11 mPa s (blue marker) or 53 mPa s (black marker). A sphere with radius r_{sphere} is centred in the cross-section of a circular channel with a relative radius of λ describing the coverage. The dashed lines show the respective levels of interfacial stress.

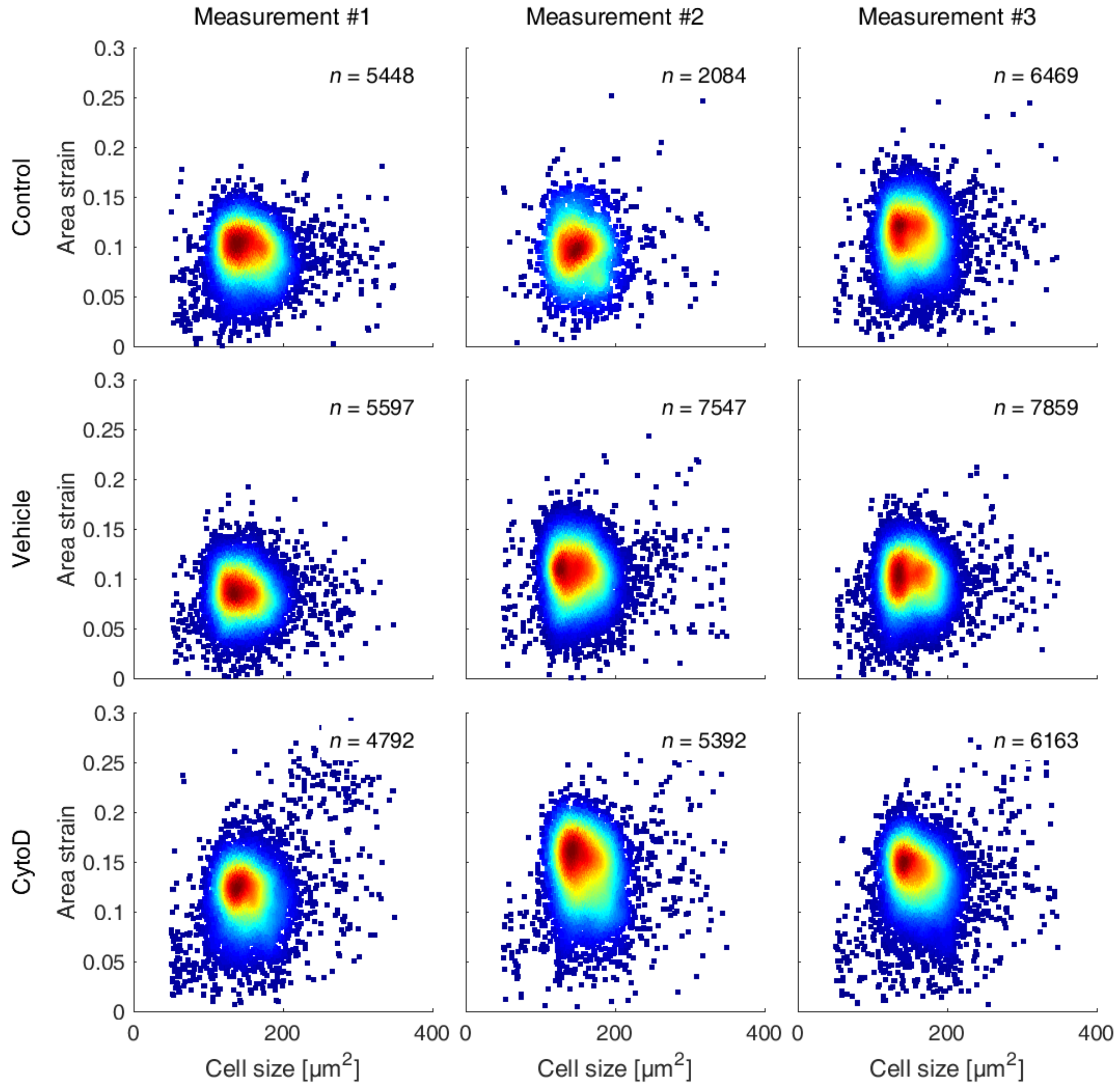

Supplementary Figure 9: Real-time deformability cytometry on HL60 cells using interface in glass cuvettes. Scatter plots of cell size and area strain inside a circular virtual channel with a cross-section of w = 14 μm in a glass cuvette with a side length of 360 μm measured at total flow rate Q_{tot} = 1015 nl s^{-1} (sample MC (114 μM), Q_{sa} = 15 nl s^{-1}; sheath PEG40000 (5 mM), Q_{sh} = 1000 nl s^{-1}). The columns show three biological measurements each in MC (control), under addition of DMSO (0.25% (v/v), vehicle), and exposure to CytoD (1 μM).

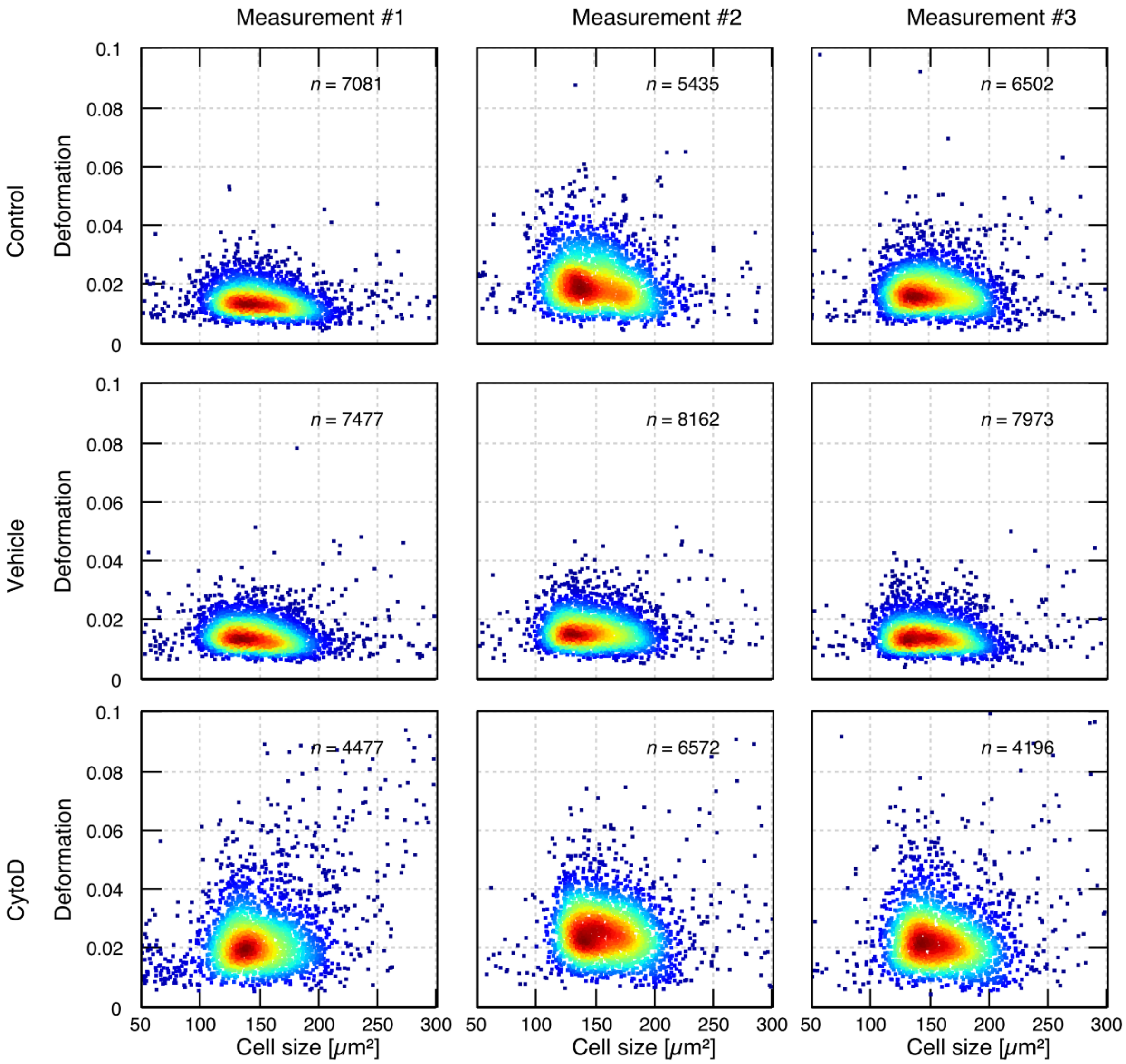

Supplementary Figure 10: Real-time deformability cytometry on HL60 cells using virtual channels in glass cuvettes. Scatter plots of cell size and deformation inside a circular virtual channel cross-section w = 20 µm) in a glass cuvette of 360 µm side length at total flow rate Q_{tot} = 1020 nl s^{-1} (sample MC (114 µM), Q_{sa} = 20 nl s^{-1}; sheath PEG40000 (5 mM), Q_{sh} = 1000 nl s^{-1}). The columns show three biological measurements each in MC (control), under addition of DMSO (0.25% (v/v), vehicle), and exposure to CytoD (1 µM).

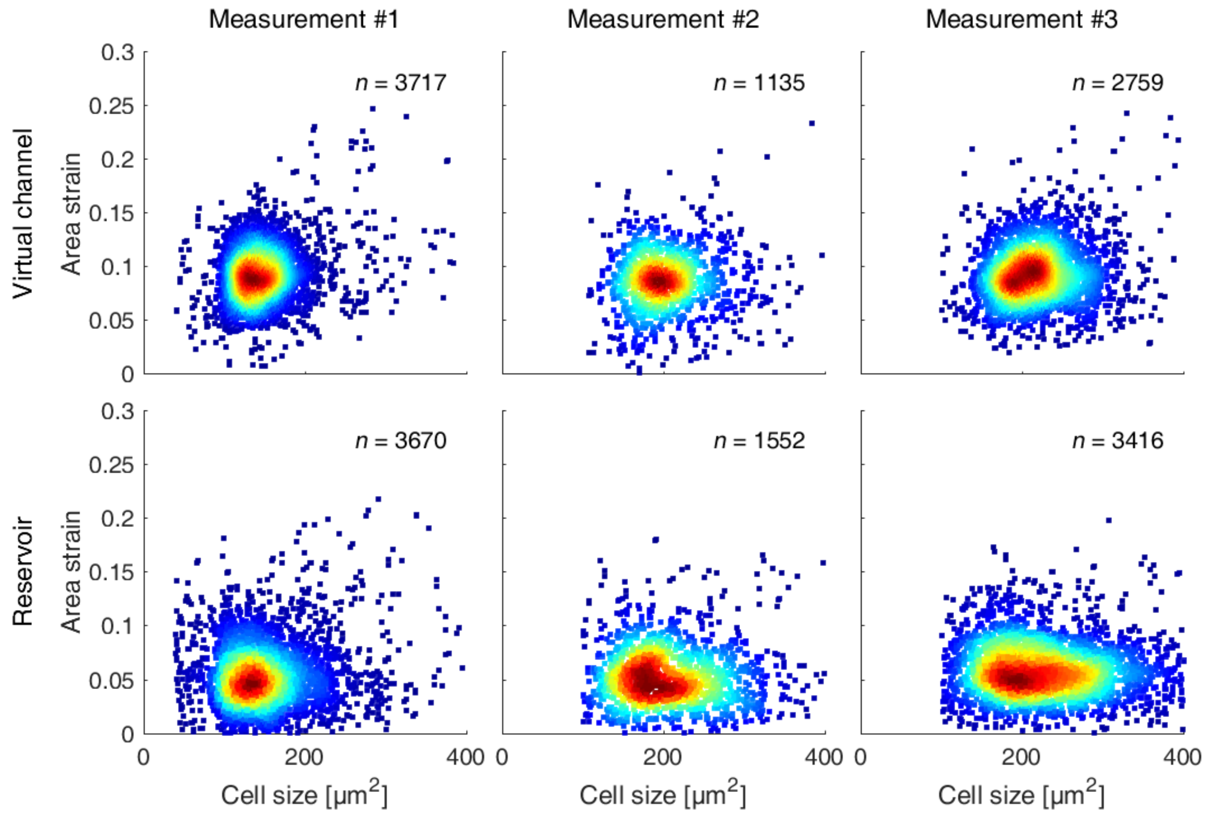

Supplementary Figure 11: Real-time deformability cytometry on HEK293T cells using virtual channels in a glass cuvette. Scatter plots of cell size and area strain inside a circular virtual channel cross-section of w = 16 µm in a glass cuvette of 360 µm side length at total flow rate Q_{tot} = 1020 nl s^{-1} (sample MC (114 µM), Q_{sa} = 20 nl s^{-1}; sheath PEG40000 (5 mM), Q_{sh} = 1000 nl s^{-1}). The columns show three biological measurements each measured in the virtual channel with where the cells are exposed to an interfacial stress 66 Pa (top) and under reservoir-like conditions (bottom).

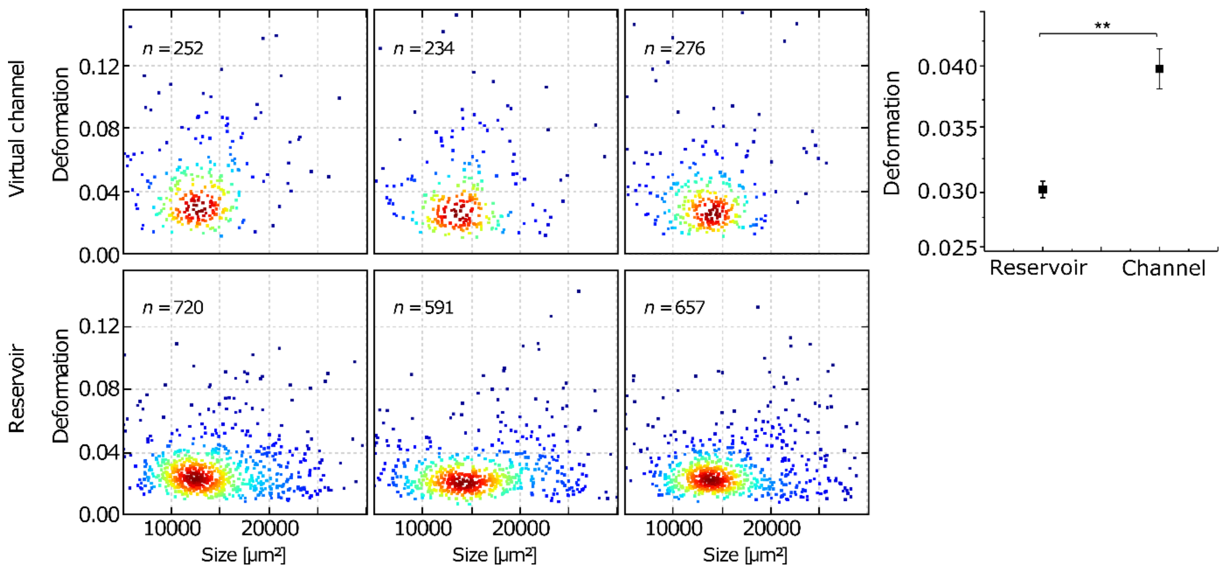

Supplementary Figure 12: Real-time deformability cytometry on HEK293T spheroids using virtual channels in a glass cuvette. Scatter plots of spheroid size and deformation inside a circular virtual channel cross-section of w = 190 µm in a glass cuvette of 360 µm side length at total flow rate Q_{tot} = 530 nl s^{-1} (sample MC (114 µM), Q_{sa} = 200 nl s^{-1}; sheath PEG40000 (5 mM), Q_{sh} = 330 nl s^{-1}). The columns show three independent biological measurements each measured in the virtual channel (top, n=762) and under reservoir-like conditions (bottom, n=1,968) at a virtual channel diameter of w = 260 µm while plot (right panel) summarizes results from statistical analysis of deformation by linear mixed models (p = 0.002). Error bars are represented by mean values ± standard error of the mean and specific p-values are listed in Supplementary Table 2 (**, p < 0.01).

Full-geometry FEM simulations				
Geometry, cross-section	Sample buffer	Q_{sa} [µl/s]	Sheath buffer	Q_{sh} [µl/s]
PDMS chip, cross-section 30 µm x 30 µm	MC (57 µM), MC (114 µM)	0.009	PEG8k (50 mM), PEG40k (2 mM), PEG40k (5mM)	0.009
		0.009		0.010
		0.009		0.020
		0.009		0.030
		0.009		0.040
		0.009		0.050
		0.009		0.060
		0.009		0.070
		0.009		0.080
		0.009		0.100
		0.009		0.200
		0.009		0.300
		0.010		0.009
		0.010		0.010
		0.010		0.025
		0.010		0.030
		0.010		0.050
		0.010		0.100
		0.010		0.200
		0.014		0.009
		0.020		0.009
		0.020		0.100
		0.023		0.009
		0.030		0.009
		0.030		0.010
		0.030		0.030
		0.030		0.100
		0.032		0.009
		0.036		0.009
		0.040		0.009
		0.040		0.012
		0.040		0.030
		0.040		0.100
		0.048		0.010
		0.048		0.120
		0.048		0.004
		0.048		0.008
		0.050		0.009
		0.050		0.010
		0.050		0.030
		0.054		0.009
		0.060		0.009
		0.060		0.012
		0.060		0.020
		0.060		0.030
		0.060		0.050
		0.060		0.100
		0.060		0.120

**Full-geometry FEM simulations**				
Geometry, cross-section	Sample buffer	Q_{sa} [µl/s]	Sheath buffer	Q_{sh} [µl/s]
PDMS chip, cross-section 30 µm x 30 µm	MC (57 µM), MC (114 µM)	0.070	PEG8k (50 mM), PEG40k (2 mM), PEG40k (5mM)	0.009
		0.070		0.030
		0.072		0.004
		0.072		0.008
		0.080		0.004
		0.080		0.008
		0.080		0.009
		0.080		0.010
		0.080		0.012
		0.080		0.030
		0.080		0.100
		0.090		0.009
		0.090		0.030
		0.090		0.090
		0.100		0.009
		0.100		0.010
		0.100		0.012
		0.100		0.020
		0.100		0.030
		0.100		0.040
		0.100		0.050
		0.100		0.060
		0.100		0.080
		0.100		0.100
		0.120		0.010
		0.120		0.050
		0.120		0.070
		0.150		0.009
		0.150		0.010
		0.150		0.030
		0.160		0.010
		0.200		0.009
		0.200		0.010
		0.200		0.020
		0.200		0.030
		0.200		0.050
		0.200		0.080
		0.240		0.080
		0.250		0.009
		0.300		0.009
		0.300		0.050
		0.350		0.050
		0.550		0.050
		0.600		0.050
		0.650		0.050
		0.700		0.050
		0.900		0.300

Full-geometry FEM simulations

Geometry, cross-section	Sample buffer	Q_{sa} [µl/s]	Sheath buffer	Q_{sh} [µl/s]
PDMS chip, cross-section 20 µm x 20 µm	MC (57 µM)	0.080	MC (57 µM)	0.004
Glass cuvette	MC (114 µM)	0.330	PEG40k (5 mM)	0.200
		0.200		0.050
		0.700		1.000
		0.200		1.000
		0.020		1.000
		0.040		2.000

FEM simulations to determine shear stress on surface

Cross-section of virtual channel	Sample buffer	Q_{sa} [µl/s]	r_{sphere} [µm]	$v_{interface}$ [cm/s]	$\dot{\gamma}$ [s^{-1}]
Circular Ø 20 µm	MC (114 µM)	0.020	6.9	1.65	148670
Circular Ø 20 µm		0.020	8.0	1.65	245230
Circular Ø 23 µm		0.040	6.9	3.30	135360
Circular Ø 190 µm		0.330	66.1	0.68	1690
Circular Ø 197 µm	53 mPa s	0.530	25.0	0.00	165
Circular Ø 197 µm		0.530	50.0		125
Circular Ø 197 µm		0.530	75.0		110
Circular Ø 197 µm		0.530	100.0		155
Circular Ø 197 µm		0.530	120.0		168
Circular Ø 197 µm		0.530	140.0		230
Circular Ø 197 µm		0.530	150.0		304
Circular Ø 197 µm		0.530	160.0		418
Circular Ø 197 µm		0.530	170.0		675
Circular Ø 197 µm		0.530	180.0		1377
Circular Ø 197 µm		0.530	187.0		3241
Circular Ø 197 µm		0.530	190.0		5879
Circular Ø 197 µm		0.530	192.0		10189
Circular Ø 197 µm		0.530	194.0		23874
Circular Ø 197 µm	11 mPa s	1.020	25.0		291
Circular Ø 197 µm		1.020	50.0		224
Circular Ø 197 µm		1.020	75.0		226
Circular Ø 197 µm		1.020	100.0		283
Circular Ø 197 µm		1.020	120.0		331
Circular Ø 197 µm		1.020	140.0		444
Circular Ø 197 µm		1.020	150.0		585
Circular Ø 197 µm		1.020	160.0		817
Circular Ø 197 µm		1.020	170.0		1304
Circular Ø 197 µm		1.020	180.0		2652
Circular Ø 197 µm		1.020	187.0		6240
Circular Ø 197 µm		1.020	190.0		11326
Circular Ø 197 µm		1.020	192.0		19618
Circular Ø 197 µm		1.020	194.0		45950
Square 20 µm x 20 µm	MC (114 µM)	0.040	6.6	0.00	116000
Rectangle 20 µm x 30 µm	MC (114 µM)	0.090	6.6	0.00	98144

Supplementary Table 1: Finite element method simulations of virtual channels. Calculations were performed for PDMS chip (top) and glass cuvette (bottom) and are used to derive the mean shear rate on cell surface. The apparent Young's modulus is calculated from an analytical model where a shear-rate dependent viscosity is assumed using power-law rheology.

control and treatment condition			figure	entity	*w* [µm]	p value	significance
Control (VC)	vs	CytoD (VC)	Fig 3	deformation	21	0.00835	**
Control (PDMS)	vs	CytoD (PDMS)	Fig 3	deformation	20	0.00097	***
Vehicle (PDMS)	vs	CytoD (PDMS)	Fig 3	deformation	20	0.00046	***
Vehicle (VC)	vs	CytoD (VC)	Fig 3	deformation	21	0.00289	**
Control (PDMS)	vs	CytoD (PDMS)	Fig 3	cell size	20	0.00076	***
Control (PDMS)	vs	Control (VC)	Fig 3	cell size	20/21	0.00197	**
Vehicle (PDMS)	vs	Vehicle (VC)	Fig 3	cell size	20/21	0.00105	**
CytoD (PDMS)	vs	CytoD (VC)	Fig 3	cell size	20/21	0.00643	**
Control (VC)	vs	Vehicle (VC)	Fig 3	cell size	21	0.01170	*
Control (VC)	vs	CytoD (VC)	Fig 3	cell size	21	0.03372	*
Control (VC)	vs	CytoD (VC)	Fig 3	Young's modulus	21	0.00923	**
Control (PDMS)	vs	CytoD (PDMS)	Fig 3	Young's modulus	20	0.00026	***
Vehicle (PDMS)	vs	CytoD (PDMS)	Fig 3	Young's modulus	20	0.01752	*
Vehicle (VC)	vs	CytoD (VC)	Fig 3	Young's modulus	21	0.01133	*
Control (VC)	vs	CytoD (VC)	Fig 5B	Young's modulus	14	0.00644	**
Vehicle (VC)	vs	CytoD (VC)	Fig 5B	Young's modulus	14	0.00055	***
Control (VC)	vs	CytoD (VC)	Fig 5V	Young's modulus	20	0.03009	*
Vehicle (VC)	vs	CytoD (VC)	Fig 5C	Young's modulus	20	0.00034	***
single HEK (VC)	vs	HEK spheroid (VC)	Fig 6C	Young's modulus	16/190	0.00110	**
HEK spheroid (VC)	vs	HEK spheroid (VC)	Fig S12	deformation	260/190	0.00220	**

Supplementary Table 2: Exact p-values determined from linear mixed models. Pairwise comparison between a set of control and treatment conditions. Measurements are carried out either in a PDMS solid wall channel (labelled PDMS) or in a liquid-liquid virtual channel (labelled VC). Width *w* refers to PDMS channel width for experiments in PDMS chips and to virtual channel width for experiments labelled VC. (*, $p < 0.05$; **, $p < 0.01$; ***, $p < 0.001$).
