# Supplementary material for: High-throughput cell and spheroid mechanics in virtual fluidic channels
Source: Nat Commun. 2020 May 4;11:2190. doi: 10.1038/s41467-020-15813-9 (PMC7198589; doi:10.1038/s41467-020-15813-9)
Supplement: Supplementary file 3 — Description of Additional Supplementary Files [file 41467_2020_15813_MOESM3_ESM.pdf]

Description of Additional Supplementary Files

File Name: Supplementary Movie 1
Description: **Long-term virtual channel stability.** Video shows virtual channel stability inside a 2 mm long channel of a poly-dimethyl siloxane chip of 28 µm x 28 µm cross-section. Virtual channel formation has been done using 57 µM methylcellulose with a sample flow rate Qsa = 120 nl s^{-1} and 5 mM polyethylene glycol 40,000 with a sheath flow rate Qsh = 70 nl s^{-1}. Scale bar is 100 µm.

File Name: Supplementary Movie 2
Description: **Virtual channel diameter adjustment.** Video shows adjustment of virtual channel diameter inside a 300 µm long channel of a poly-dimethyl siloxane chip of 30 µm x 30 µm cross-section. Virtual channel width is modified between 5 µm and 30 µm adjusting sheath and sample flow rate between 4 nl s^{-1} and 100 nl s^{-1} using 60 mM polyethylene glycol 8,000 and 57 µM methylcellulose, respectively. Video has been sped up 50 times. Scale bar is 30 µm.
