# Supplementary material for: High-throughput cell and spheroid mechanics in virtual fluidic channels
Source: Nat Commun. 2020 May 4;11:2190. doi: 10.1038/s41467-020-15813-9 (PMC7198589; doi:10.1038/s41467-020-15813-9)
Supplement: Supplementary file 6 — Reporting Summary [file 41467_2020_15813_MOESM6_ESM.pdf]

nature research

Corresponding author(s): Oliver Otto

Last updated by author(s): Mar 17, 2020

Reporting Summary

Nature Research wishes to improve the reproducibility of the work that we publish. This form provides structure for consistency and transparency in reporting. For further information on Nature Research policies, see Authors & Referees and the Editorial Policy Checklist.

Statistics

For all statistical analyses, confirm that the following items are present in the figure legend, table legend, main text, or Methods section.

n/a	Confirmed	
☐	☒	The exact sample size (*n*) for each experimental group/condition, given as a discrete number and unit of measurement
☐	☒	A statement on whether measurements were taken from distinct samples or whether the same sample was measured repeatedly
☐	☒	The statistical test(s) used AND whether they are one- or two-sided<br>*Only common tests should be described solely by name; describe more complex techniques in the Methods section.*
☒	☐	A description of all covariates tested
☐	☒	A description of any assumptions or corrections, such as tests of normality and adjustment for multiple comparisons
☐	☒	A full description of the statistical parameters including central tendency (e.g. means) or other basic estimates (e.g. regression coefficient) AND variation (e.g. standard deviation) or associated estimates of uncertainty (e.g. confidence intervals)
☐	☒	For null hypothesis testing, the test statistic (e.g. *F*, *t*, *r*) with confidence intervals, effect sizes, degrees of freedom and *P* value noted<br>*Give P values as exact values whenever suitable.*
☒	☐	For Bayesian analysis, information on the choice of priors and Markov chain Monte Carlo settings
☒	☐	For hierarchical and complex designs, identification of the appropriate level for tests and full reporting of outcomes
☒	☐	Estimates of effect sizes (e.g. Cohen's *d*, Pearson's *r*), indicating how they were calculated

Our web collection on statistics for biologists contains articles on many of the points above.

Software and code

Policy information about availability of computer code

Data collection	Data acquisition, image analysis, and the control of the syringe pump are performed by using the ShapeIn2 software (version 2.0.5, Zellmechanik Dresden).
Data analysis	Data acquisition, image analysis, and the control of the syringe pump are performed by using the ShapeIn2 software (version 2.0.5, Zellmechanik Dresden). Data analysis has been done using Comsol Multiphysics (version 5.4, Comsol Group, finite element method simulations) and ShapeOut (version 0.8.7, Zellmechanik Dresden) as well as Matlab (version R2017a, Mathworks) for statistical data analysis) as outlined in the Methods section. The Comsol Multiphysics mesh files and the Matlab script are available on request. ShapeOut is available as open source software.

For manuscripts utilizing custom algorithms or software that are central to the research but not yet described in published literature, software must be made available to editors/reviewers. We strongly encourage code deposition in a community repository (e.g. GitHub). See the Nature Research guidelines for submitting code & software for further information.

Data

Policy information about availability of data

All manuscripts must include a data availability statement. This statement should provide the following information, where applicable:

- Accession codes, unique identifiers, or web links for publicly available datasets
- A list of figures that have associated raw data
- A description of any restrictions on data availability

The datasets generated in this study are available from the corresponding author upon reasonable request. The file formats of the raw rata are AVI for videos, TDMS and RTDC (HDF5) for RT-DC data, and MPH for Comsol Multiphysics simulations.

Field-specific reporting

Please select the one below that is the best fit for your research. If you are not sure, read the appropriate sections before making your selection.

☒ Life sciences ☐ Behavioural & social sciences ☐ Ecological, evolutionary & environmental sciences

For a reference copy of the document with all sections, see nature.com/documents/nr-reporting-summary-flat.pdf

Life sciences study design

All studies must disclose on these points even when the disclosure is negative.

Sample size	Sample size for experiments on cell lines and spheroids was determined by monitoring median deformation value. In general, sample sizes of approximately n=1,000 (cells) and n=100 (spheroids) have been proven to be sufficient for a stable statistic.
Data exclusions	For data analysis a convex contour filter has been applied.<br>Quantification of cell deformation relies on cellular perimeter and area and requires fitting a contour to each cell. Quality of the fit is determined by comparing the area inside the contour to the actual cell area. Data has been excluded if both parameters deviate more than 30% (spheroids) and more than 5% (cell line).<br>Cell data exclusion criteria has been established in previous works. For spheroids we have checked the relevance of the contour filter on the elastic modulus for deviations between contour area and actual area for values between 5% and 30% and found a standard deviation of 0.02kPa for 0.14kPa Young's modulus.
Replication	Mechanical cell / spheroid measurements have been carried out for biological replicates and/ or on separate days. All findings could be replicated at least three times.
Randomization	Not relevant as experiments have been carried out comparing cell / spheroid samples with and without chemical stimulus. For a given replicate the vial was split into two, where one was treated with the compound. In this sense the samples have been randomized. This study focuses on the introduction of a new technology and not on the nature of the stimulus.
Blinding	Not relevant as experiments have been carried out on cell lines / spheroids were a known chemical stimulus was tested regarding alterations in mechanical properties. The effect of the chemical stimulus was known and we introduced a new technology to study this effect.

Reporting for specific materials, systems and methods

We require information from authors about some types of materials, experimental systems and methods used in many studies. Here, indicate whether each material, system or method listed is relevant to your study. If you are not sure if a list item applies to your research, read the appropriate section before selecting a response.

Materials & experimental systems

n/a	Involved in the study	
☒	☐	Antibodies
☐	☒	Eukaryotic cell lines
☒	☐	Palaeontology
☒	☐	Animals and other organisms
☒	☐	Human research participants
☒	☐	Clinical data

Methods

n/a	Involved in the study	
☒	☐	ChIP-seq
☒	☐	Flow cytometry
☒	☐	MRI-based neuroimaging

Eukaryotic cell lines

Policy information about cell lines

Cell line source(s)	HL60 cell lines as courtesy of Don and Ada Olins. They established the cell line.
Authentication	Identification of HL60 cells was carried out by Don and Ada Olins who created the cell line. HEK293T cells have not been authenticated. Since we demonstrate the capability of virtual channels to perform cell and tissue studies an authentication is not essential for the experiments.
Mycoplasma contamination	Cell lines were tested negative for Mycoplasma contamination as stated in the Methods section.
Commonly misidentified lines (See ICLAC register)	No commonly misidentified cell line has been used.
